# Analysis of 25 surgical cases of thymic neuroendocrine tumors and thymic carcinoma

**DOI:** 10.1186/s13019-024-02723-w

**Published:** 2024-04-16

**Authors:** Kensuke Midorikawa, So Miyahara, Nanako Nishino, Yuichirou Ueda, Ryuichi Waseda, Takeshi Shiraishi, Toshihiko Sato

**Affiliations:** Department of General Thoracic, Breast and Pediatric Surgery, Fukuoka University School of Medicine and Hospital, 7-45-1 Nanakuma, Jonan-ku, Fukuoka city, 814-0180 Fukuoka Japan

**Keywords:** Thymus, Mediastinal tumor, Tumor, Thoracoscopy/VATS

## Abstract

**Background:**

The purpose of this study was to evaluate the clinicopathological characteristics of patients who underwent surgical resection for thymic neuroendocrine tumors (TNET) or thymic carcinoma.

**Methods:**

In this study, we retrospectively evaluated the clinicopathological characteristics of our surgical patients at Fukuoka University Hospital from January 1995 to December 2018.

**Results:**

There were nine cases of TNET and 16 cases of thymic carcinoma. Regarding the pathological type, the TNET group included three atypical carcinoid cases, two large cell neuroendocrine tumor cases, two small cell carcinoma cases, and two other cases. The thymic carcinoma group included 15 squamous carcinoma cases and one case of adenosquamous carcinoma. Based on the Masaoka-Koga staging system, six TNET cases and 11 thymic carcinoma cases were stage III or IV. The complete resection rate was 77% in the TNET group and 81% in the thymic carcinoma group. Additional chemotherapy and/or radiotherapy was performed in five cases of TNET and 11 cases of thymic carcinoma. The five-year survival rate and five-year disease-free survival rate were 87.5% and 75.0% in the TNET group and 58.9% and 57.1% in the thymic carcinoma group, respectively, with no significant difference between the two groups (*P* = 0.248 and *P* = 0.894, respectively). In the univariate analysis, complete resection was a statistically significant prognostic factor (*P* = 0.017).

**Conclusion:**

In this study, no difference in prognosis was observed between TNET and thymic carcinomas. To understand the characteristics of these tumors, further case accumulation and multicenter clinical studies are needed. (243words)

## Introduction

Thymic neuroendocrine tumors (TNET) and thymic carcinoma are rare thymic malignancies, with incidences of 2–3% and 15–20%, respectively [1, 2]. According to the fourth and latest fifth edition World Health Organization (WHO) Classification of Tumours of Lung, Pleura, Thymus and Heart, TNET are introduced as a single major category, although they used to belong to different categories in the third edition [3–5]. The comparative clinical characteristics of TC and TNET have not been well studied. As such, it is currently unclear whether these two distinct histological subtypes will benefit from existing customized treatment strategies in real-world clinical settings. In this study, we reviewed cases of TNET and thymic carcinoma that were resected at our department to analyze their clinical features, prognosis, and the significance of multidisciplinary treatment with surgical therapy. The purpose of researching and comparing thymic carcinoma and thymic neuroendocrine tumors is to understand the distinct clinical outcomes, survival rates, and factors influencing the progression of these rare thymic malignancies. We believe that our clinical data can contribute to data accumulation so as to understand the biological properties of these tumors.

## Patients and methods

### Patient and data Collection

A retrospective chart review was performed to identify patients who underwent thymectomy for TNET or thymic carcinoma at our department from January 1995 to December 2018. We looked over the background (age and sex), intraoperative and perioperative data (surgical approach, combined resected organs, completeness of resection, neoadjuvant therapy, and adjuvant therapy), pathological findings (histology, Masaoka stage, maximum specimen diameter, and lymph node metastasis), and follow-up data (presence or absence of recurrence, recurrence site, recurrence treatment, and cause of death). The histological type was determined according to the latest WHO classification, and staging was performed for all patients according to the Masaoka-Koga system [6]. Preoperative chemotherapy and/or radiotherapy was planned for patients who were suspected to have infiltration of the surrounding structures. The resection status was classified into three groups: R0 (complete resection as determined macroscopically and microscopically), R1 (microscopical incomplete resection), and R2 (macroscopical incomplete resection). Postoperative chemotherapy and/or radiotherapy was planned for those patients with recurrence or incomplete resection, as well as for those with a high risk of recurrence, such as a close surgical margin.

After discharge, all the patients were followed up. Chest computed tomography and blood tumor markers were reviewed every 3 months in the first year, every 6 months in the next 4 years, and then once a year after 5 years of surgery. Positron emission tomography - computed tomography, cranial magnetic resonance imaging, and whole body bone scans were reviewed as needed. The follow-up time was calculated from the date of surgery, and the last follow-up date was December 31, 2022.

The pattern of recurrence after resection was classified according to the protocol of the International Thymic Malignancy Interest Group [7].

All survival rates were calculated from the time of resection. The overall survival (OS) rate was defined as the time from surgery to death from any cause. Progression-free survival (PFS) was defined as the time from surgery to clinical progression or death.

All specimens were fixed in 10% formalin, and 4 μm sections were routinely stained with hematoxylin and eosin. For immunohistochemical studies, synaptophysin, chromogranin, and CD56 were used as neuroendocrine markers to evaluate neuroendocrine differentiation of the thymic neoplasm. CD5, c-kit, and p40 were used as thymic carcinoma markers to identify thymic carcinoma.

### Statistical analysis

Differences between the two groups were examined using the t test and chi-square test. Survival curves were calculated by the Kaplan‒Meier method, and *P* < 0.05 was considered significant by the log-rank test. Univariate analysis by Cox proportional hazards analysis was used to evaluate prognostic factors. StateMate V (ATOMOS: JAPAN) was used for all statistical analyses.

## Results

### Patient characteristics

There were 25 patients who were histopathologically diagnosed with TNET or thymic carcinoma. They included nine cases of TNET (including two cases with neuroendocrine tumor components) and 16 cases of thymic carcinoma. Their backgrounds are summarized in Table 1. TNET cases are summarized in Table 2, and cases of thymic carcinoma are summarized in Table 3. The median follow-up period in all cases was 1126 days (9–4939 days). The average age at the time of surgery was 60.0 (45–83) years for TNET and 61.6 (39–82) years for thymic carcinoma. TNET were comparatively larger than thymic carcinoma, as the average tumor diameter was 61.8 mm (34–104 mm) in TNET and 53.0 mm (20–100 mm) in thymic carcinoma. Six cases (67%) of TNET and 11 cases (69%) of thymic carcinoma were Masaoka stage III or IV.

**Table 1:** Characteristics of study participants

	TNET *N* = 9	TC *N* = 16	*P*-value
Sex			
Male Female	8(32) 1(4)	8(32) 8(32)	0.051
Age, years			
Mean/ Range	60.0 / 45–83	61.6 / 39–82	0.737
Histology	LCNEC 3 (33) Ac 2 (22) SCC 2(22) *Other 2(22)	Sq 15 (93) AdSq 1 (7)	-
Tumor size. mm			
Mean/ Range	61.8 / 34–104	53 / 20–100	0.527
Masaoka stage			
I II III IV	0(0) 3(33) 3(33) 3(33)	1 (6) 4 (25) 7 (43) 4 (25)	0.800
Preoperative therapy			
No Yes	7(77) 2(22)	14(87) 2 (12)	0.527
Combined resection			
Yes No	6(66) 3(33)	13(81) 3(18)	0.412
Lymph node metastases			
N0 N1, 2	8 (88) 1(11)	13 (81) 3(18)	0.617
Resection status			
R0 R1,2	8 (88) 1(11)	13(81) 3(18)	0.617
Postoperative therapy			
No Yes	5 (55) 4(44)	5(31) 11 (68)	0.233
Recurrence			
No Yes	4 (44) 5 (55)	11 (68) 5 (31)	0.233

* “Other” includes: Thymic carcinoma with neuroendocrine carcinoma components (1), poorly differentiated thymic neuroendocrine carcinoma (1)

TNET, thymic neuroendocrine tumors; TC, thymic carcinoma; LCNEC, large cell neuroendocrine carcinoma; Ac, atypical carcinoid; SCC, small cell carcinoma; Sq, squamous cell carcinoma; AdSq, adenosquamous carcinoma

**Table 2:** Clinical findings in patients with TNET

	Age (years) /Sex	Histology	Tumor size (mm)	Preoperative therapy			Resection status	Combined resection	Masaoka stage	Postoperative therapy		Relapse site	Prognosis (Survival time, days)
				CT	RT (Gy)	Effect				CT	RT (Gy)		
1	58 M	Combined*	45	-	-		R2	-	4a	-	-	-	A(51)
2	51 M	LCNEC	78	-	-		R0	L, PC, PN	2	-	-	Local	A(672)
3	83 M	Ac	104	-	-		R0	PC	2	-	-	-	A(233)
4	68 M	LCNEC	75	-	-		R0	L PC	4a	ADOC	PRT	Distant	D(1410)
5	45 F	Ac	62	CDDP + VP-16	40	PR	R0	PC	3	SST, Ev	-	Distant	A(2912)
6	61 M	Poorly	65	-	-		R0	-	2	CDDP + VP-16	-	-	A(2994)
7	70 M	Ac	90	-	-		R0	BCV, PC	4b	-	-	Local	A(3587)
8	54 M	SCC	65	-	-		R0	L	3	CDDP + VP-16	50 Gy	Distant	D(3630)
9	50 M	SCC	34	CDDP + VP-16 + ADM	45	PR	R0	-	3	-	-	-	A(4870)

*Combined: Thymic carcinoma with neuroendocrine carcinoma components

TNET, thymic neuroendocrine tumors; M, male; F, female; LCNEC, large cell neuroendocrine carcinoma; Ac, atypical carcinoid; SCC, small cell carcinoma; Poorly, poorly differentiated thymic neuroendocrine carcinoma; CT, chemotherapy; RT, radiotherapy; CDDP, cisplatin; VP-16, etoposide; ADM, doxorubicin hydrochloride; PR, partial response; PRT, palliative radiotherapy; ADOC, doxorubicin hydrochloride + cisplatin + vincristine sulfate + cyclophosphamide; L, lung; PC, pericardium; PN, phrenic nerve; BCV, brachiocephalic vein; SST, somatostatin; Ev, everolimus; A, alive; D, dead

**Table 3:** Clinical findings in patients with TC

	_Age (years)/ Sex_	_Histology_	_Tumor_ _size_ _(mm)_	_Preoperative therapy_			_Resection_ _status_	_Combined resection_	_Masaoka stage_	_Postoperative therapy_		_Relapse site_	_Prognosis_ _(Survival time, days)_
				_CT_	_RT_ _(Gy)_	_Effect_				_CT_	_RT_ _(Gy)_		
_1_	_48 M_	_Adsq_	_35_	_−_	_−_		_R0_	_L, PN, RLN, SA_	_3_	_−_	_−_	_−_	_A(9)_
_2_	_39 M_	_Sq_	_78_	_−_	_−_		_R0_	_L, PC, PN_	_3_	_−_	_60_	_Distant_	_D(412)_
_3_	_72 M_	_Sq_	_80_	_−_	_−_		_R0_	_L_	_2_	_−_	_50_	_−_	_A(449)_
_4_	_65 M_	_Sq_	_28_	_−_	_−_		_R0_	_−_	_4b_	_−_	_−_	_Distant_	_A(544)_
_5_	_66 F_	_Sq_	_35_	_−_	_−_		_R1_	_BCA, BCV,_ _SVC, L, SA,_ _SV, PN, VN_	_3_	_−_	_54_	_−_	_A(647)_
_6_	_56 M_	_Sq_	_75_	_CDDP+GEM, CBDCA+ADM_	_50_	_SD_	_R1_	_BCV, L, PC_	_3_	_−_	_−_	_Local_	_D(804)_
_7_	_53 F_	_Sq_	_20_	_−_	_−_		_R0_	_−_	_2_	_−_	_54_	_−_	_A(849)_
_8_	_76 F_	_Sq_	_35_	_−_	_−_		_R2_	_L_	_4a_	_CBDCA+PTX, S−1_	_−_	_Local_	_D(1076)_
_9_	_82 F_	_Sq_	_39_	_−_	_−_		_R0_	_PC_	_3_	_−_	_−_	_Distant_	_D(1176)_
_10_	_78 M_	_Sq_	_66_	_−_	_−_		_R0_	_PC, SVC_	_3_	_−_	_60_	_−_	_A(1194)_
_11_	_59 M_	_Sq_	_70_	_−_	_−_		_R0_	_PC, L_	_2_	_−_	_−_	_−_	_A(1441)_
_12_	_66 F_	_Sq_	_100_	_CDDP+ADM+VCR+CPA, CBDCA+PTX_	_−_	_SD_	_R0_	_L_	_4b_	_CBDCA+PTX, CDDP+VP−16_	_−_	_Local_	_D(1474)_
_13_	_49 M_	_Sq_	_60_	_−_	_−_		_R0_	_L_	_2_	_−_	_60_	_−_	_A(1896)_
_14_	_68 F_	_Sq_	_20_	_−_	_−_		_R0_	_−_	_1_	_−_	_50_	_−_	_A(3293)_
_15_	_53 F_	_Sq_	_34_	_−_	_−_		_R0_	_BCV_	_3_	_CBDCA+nab−PTX, DOC_	_50_	_Distant_	_D(3713)_
_16_	_56 F_	_Sq_	_80_	_−_	_−_		_R0_	_L, PL_	_4b_	_−_	_RT(unknown)_	_−_	_A(4939)_

TC, thymic carcinoma; M, male; F, female; AdSq, adenosquamous carcinoma; Sq, squamous carcinoma; CT, chemotherapy; RT, radiotherapy; CDDP, cisplatin; GEM, gemcitabine; CBDCA, carboplatin; ADM doxorubicin hydrochloride; VCR, vincristine sulfate; CPA, cyclophosphamide; PTX, paclitaxel; S-1, tegafur/gimeracil/oteracil; SD, stable disease; L, lung; PN, phrenic nerve; RLN, recurrent laryngeal nerve; SA, subclavian artery; PC, pericardium; BCA, brachiocephalic artery; BCV, brachiocephalic vein; SVC: superior vena cava, SV, subclavian vein; VN, vagus nerve; PL, pleura; VP-16, etoposide; nab-PTX, albumin-bound PTX; A, alive; D, dead

### Preoperative therapy

In the TNET group, we preoperatively added chemoradiotherapy to two patients. In the thymic carcinoma group, we added preoperative chemoradiotherapy for one patient and chemotherapy for one patient. These patients were mainly treated with platinum-based chemotherapy. In particular, TNET were treated with cisplatin (CDDP)/etoposide (VP-16) chemotherapy following the regimen used for small cell lung cancer. TNET Case 5 was an atypical carcinoid diagnosed as MEN type 1 with parathyroid tumor, pituitary tumor, and pancreatic tumor. The patient underwent one course of CDDP + VP-16 and 40 Gy radiotherapy and showed a partial response (PR). TNET Case 9 showed a PR after preoperative chemoradiotherapy for small cell carcinoma. Thymic carcinoma Case 6 underwent preoperative chemotherapy for suspicious invasion into the aorta and brachiocephalic vein and showed stable disease (SD). Thymic carcinoma Case 12 underwent chemotherapy for possible infiltration into the aorta and was identified as an SD, requiring salvage surgery.

### Surgical treatment

Sternotomy was performed in all nine cases of TNET. For thymic carcinoma, sternotomy was performed in 11 cases, posterolateral thoracotomy was performed in one case and sternotomy and intercostal thoracotomy was performed in one case. Thoracoscopic surgery was performed in three cases. Combined resection of adjacent organs was performed in six cases (67%) of TNET and 13 cases (81%) of thymic carcinoma, with no statistically significant difference. In all cases, no operative (30-day) mortality occurred, and postoperative morbidity rate was 12% (one case of patients, pleural effusion, gastrointestinal bleeding, atelectasis out of 25 cases).

### Pathological findings

Regarding the pathological type, the TNET group included three atypical carcinoid cases, two large cell neuroendocrine tumor cases, two small cell carcinoma cases, and two other cases. The thymic carcinoma group included 15 squamous carcinoma cases and one case of adenosquamous carcinoma. Lymph node metastasis was positive in one case (11%) of TNET and three cases (18%) of thymic carcinoma. Complete resection was achieved in eight cases (89%) of TNET and 13 cases (81%) of thymic carcinoma. In the TNET group, one patient had R2 residual disease with pleural dissemination. In the thymic carcinoma group, two cases were R1 with a positive surgical margin, and one case was R2 with pleural dissemination.

### Postoperative treatment

Four cases of TNET and 10 cases of thymic carcinoma needed additional postoperative treatment. For TNET, we added adjuvant chemotherapy in two patients and adjuvant chemoradiotherapy in one patient, as shown in Table 2. In Case 5 of TNET, Sandostatin® for recurrence was discontinued due to the side effect of diarrhea, followed by everolimus for six months, which was again discontinued for financial reasons. For thymic carcinoma, radiotherapy was given postoperatively in eight cases, and chemotherapy was given postoperatively in one case and at the time of recurrence in two cases, as shown in Table 3.

### Recurrence and survival

**Fig. 1:**
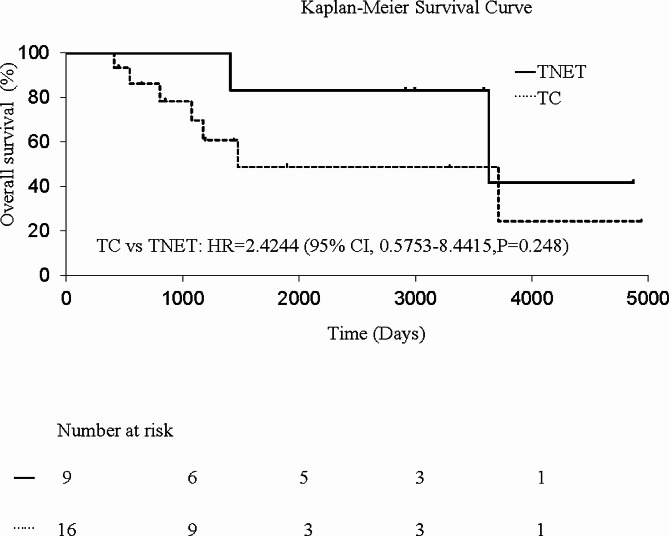
Kaplan‒Meier curves comparing the survival of patients with TNET and TC. TNET, thymic neuroendocrine tumors; TC, thymic carcinoma; HR, hazard ratio; CI, confidence interval

**Fig. 2:**
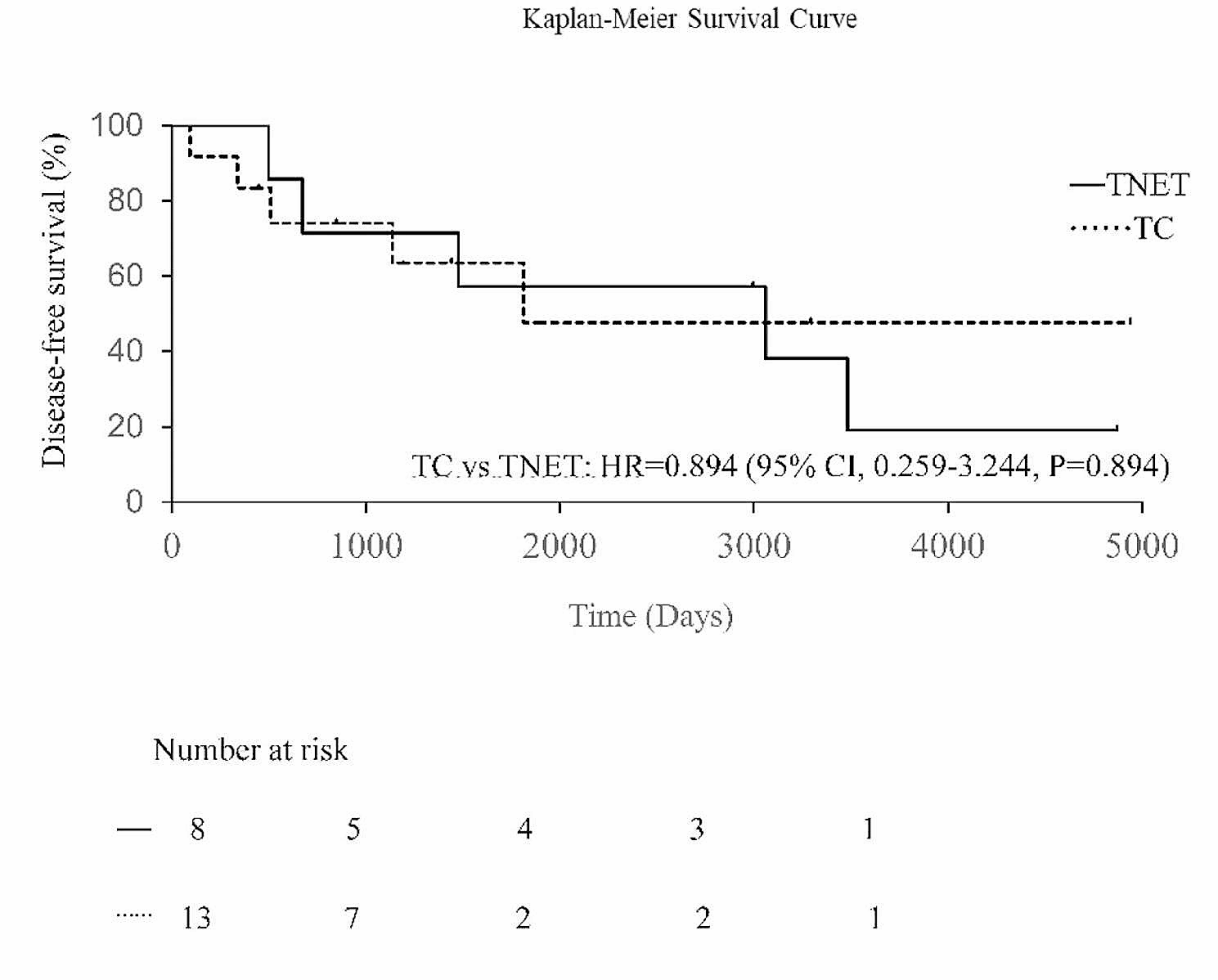
Kaplan‒Meier curves comparing the disease-free survival after complete resection in patients with TNET and TC. TNET, thymic neuroendocrine tumors; TC, thymic carcinoma; CI, confidence interval

Some patients developed recurrence even after radical surgery, as described below. In TNET and thymic carcinoma, local recurrence was observed in two cases and one case, respectively, and distant metastasis was observed in three cases and four cases, respectively. Survival curves of TNET and thymic carcinoma are shown in Figs. 1 and 2. The five-year survival rate and five-year disease-free survival rate were 87.5% and 75.0% in TNET and 58.9% and 57.1% in thymic carcinoma, respectively, with no significant difference between the two tumor groups. In univariate analysis, complete resection was found to be a significant prognostic factor, while pathological type, Masaoka stage, and tumor size showed no significance (Table 4).

**Table 4:** Univariate analysis of parameters influencing OS

	HR (95% CI)	*P*-value
Gender (Male vs. Female)	1.147(0.306–4.316)	0.835
Age (>60 vs. ≦ 60)	0.467 (0.085–1.681)	0.2018
Histology (TNET vs. TC)	2.424 (0.575–8.4415)	0.2488
Tumor size (≧50mm vs<50)	1.285(0.328–5.268)	0.6982
Masaoka-Koga stage (I, II vs. III, IV)	0 (0.047–1.206)	0.083
Preoperative therapy (yes vs. no)	1.108(0.237–5.151)	0.897
Combined resection (yes vs. no)	0.372(0.103–2.142)	0.330
Lymph node meta (N0 vs. N1, 2)	1.069 (0.2258–5.0589)	0.9330
Resection status (R0 vs. R1, 2)	0.193(0.001–0.515)	0.017
Postoperative therapy (yes vs. no)	1.545(0.398–6.354)	0.510

OS, overall survival; TNET, thymic neuroendocrine tumors; TC, thymic carcinoma; HR, hazard ratio

## Discussion

In the NCCN guidelines, TNET belongs to neuroendocrine tumors and is treated in a different protocol than thymic carcinoma [8, 9]. In both TNET and thymic carcinoma, the grade of malignancy is determined based on histologic features. However, it is not easy to compare the degree of malignancy, as both groups include several histologic types. Suster et al. investigated 60 cases of malignant thymic tumors and found that squamous cell carcinoma showed low-grade histology, and small cell/neuroendocrine carcinoma showed high-grade histology [10]. On the other hand, TNEThas a pathological variety, including typical carcinoids, atypical carcinoids, large cell neuroendocrine tumors, and small cell carcinomas. They are classified as follows, based on histologic features such as tumor growth pattern, cell atypia, and mitosis number: typical carcinoids and atypical carcinoids, and both large cell neuroendocrine tumors and small cell carcinomas are classified as low grade, intermediate grade, and high grade, respectively [11]. High-grade TNET, such as large cell neuroendocrine tumors and small cell carcinomas, often develop infiltration and cause lymph node metastasis. Therefore, they generally have a poor prognosis similar to that of lung primary neuroendocrine carcinomas [12, 13].

In our investigation encompassing nine cases of thymic endocrine tumors and 16 cases of thymic carcinoma, no statistically significant differences were observed in patient backgrounds. However, it is noteworthy that a male predominance was observed, consistent with previous reports. This is in clear contrast to other neuroendocrine tumors, where the incidence in males and females is usually more equal.

In this study, there was no statistically significant difference between their five-year survival rates. Although this result does not agree with previous reports [10, 11], some studies regarding surgical cases show similar results to ours. Table 5 summarizes reports comparing the prognosis of TNET and thymic carcinoma [14–20]. Filosso et al. investigated surgical cases of TNET and thymic carcinoma and found that the five-year survival rates were 68% and 60% in TNET and thymic carcinoma, respectively, with no difference in prognosis [16]. It is not easy to simply compare the previous reports, as each report on thymic malignancy includes different histological types, with different diagnostic or treatment protocols. The variety of histological types, as well as the rarity of TNET and thymic carcinomas, make it difficult to understand their biologicalnature.

**Table 5:** Summary of studies related to TC and TNET

Author (published year)	No.	Pathology	Surgery case	R0 Resection rate(%)	Survival
our study	25	TC:16[Sq(*n* = 15),Adsq(*n* = 1)] TNET:9[Ac(*n* = 3),LCNEC(*n* = 2),SCC(*n* = 2), other(*n* = 2)]	100%(25/25)	TC:81 TNET:88	5yOS/5yDFS TC:58.9/ 57. % TNET:87.5/ 75%
Kondo [15] (2003)*	1320	TC:186 [Sq1(*n* = 115),und(*n* = 27),SCC(*n* = 16),Ad(*n* = 5) ,Adsq(*n* = 4),other(*n* = 7)] TCD:41 [Tc or Ac(*n* = 41)] Thy:1093	TC:71.9% TCD:92.5% Thy:97%	NA	5yOS TC:84.4% TCD:84.4% Thy:94.4%
Benny [16] (2015)	229	TC:176 TNET:53	TC + TNET Resection 93%/ Debulking7%	NA	mOS TC:85 m TNET:117 m
Filosso [17] (2016)	1247	TC:1042[Sq79%,other21%] TNET:205[Tc(*n* = 49),Ac(*n* = 71), LCNECorSCC(*n* = 49)]	100% (1227/1227)	TC:60 TNET:54	5yOS/10yOS/5yRFS TC:60/40/35% TNET:68,1/39,5/34%
Zhao [18] (2017)	343	TC:287 TCD:56 (Tc or Ac)	TC:90.6% TCD:86.4%	TC:45.6 TCD:53.6	5yOS/5yDFS TC:60.7/41.1% TCD:80.7/37.6%
Wen [19] (2018)	3947	TC:886 [well8.1%,Mod13.6%,Poor64.6%,Und13.6%] TNET:293 [well41.5%,Mod25.8,%Poor21.4%,Und11.3%] Thy:2788	TC:58.7% TNET:66.9% Thy:78.2%	NA	TC: NA TNET: mCSS82.9 m, mOS101.9 m
Song [20] (2019)	362	TC:240 TNET:122	TC + TNET Surgery of primary site 73.2%	NA	MST TC:92 m TNET:52 m
Bakhos [21](2020)	1489	TC:80.2% TNET:19.8%	TC:55.3% TNET:58.3%	NA	5yOS TC:52% TNET:62%

*SCC is included in the TC Group in this paper

TC, thymic carcinoma; Sq, squamous cell carcinoma; AdSq, adenosquamous carcinoma; TNET, thymic neuroendocrine tumors; Ac, atypical carcinoid; LCNEC, large cell neuroendocrine carcinoma; SCC, small cell carcinoma; undifferentiated carcinoma; TCD, thymic carcinoid; Thy, thymoma; Tc, atypical carcinoid; well, well differentiated; Mod, moderately differentiated; Poor, poorly differentiated; Und, undifferentiated; NA, not aveilable; OS, overall survival; DFS, disease-free survival; mOS, median overall survival; RFS; mCSS, median cancer-specific survival; MST, median survival time

Our results showed that TNET tended to exhibit a better prognosis than thymic carcinoma, despite the absence of statistical significance. (Fig. 1) This observation could be attributed not only to the inclusion of low-grade tumors, such as typical carcinoid or atypical carcinoid, but also to the high rate of complete resection in the TNET group. In fact, in our study, the TNET group included only three cases of atypical carcinoid as low-grade tumors. Univariate analysis showed that complete resection was a possible prognostic factor. According to past reports, possible prognostic factors of TNET include the pathological type, surgical indication, Masaoka stage, complete resection, tumor size, lymph node metastasis, and distant metastasis [1, 16, 21–26]. In particular, complete resection has been reported to be a strong prognostic factor [16, 21, 22, 24]. In this study, the subjects included only surgical cases, and most of them underwent complete resection. Especially for TNET, complete resection was performed in eight out of nine cases, which included six cases requiring extended resection, as shown in Table 2. We assumed that the high rate of complete resection led to comparatively good outcomes, although there were many high-grade TNET. For instance, in TNET Case 9, the patient underwent complete resection and survived longer than 10 years, even though the histological type was small cell carcinoma. There are few past reports on surgical cases of high-grade TNET. Hamaji et al. investigated 21 surgical cases of TNET and reported that the five-year survival rate was 64.6% [23]. The results of our study were comparable to their results. Among pulmonary neuroendocrine tumors, small cell lung carcinoma grows rapidly and develops quickly to lymph node metastasis and distant metastasis. Hence, surgical indications are limited only for some localized small cell lung cancers [27]. On the other hand, some aggressive TNET show a comparatively longer prognosis after surgical resection. Even in high-grade TNET, surgical treatment, especially complete resection, can possibly extend the prognosis, unlike small cell lung carcinoma. Therefore, surgical treatment should be taken into consideration, even if extensive surgery is needed.

Even in cases of complete resection, distant metastasis was likely to occur if the Masaoka stage was III or higher in these tumors (Tables 2 and 3). It suggested the importance of tumor control through systemic therapy. In our department, additional treatment is often performed before and/or after surgery for either high-grade TNET, advanced stage, incomplete resection, or postoperative recurrence. As there is no unified protocol regarding additional perioperative treatment for TNET, each institution has to decide on their indications for perioperative treatment. In fact, some reports conclude that preoperative chemotherapy reduces tumor size and leads to complete resection. Additionally, there is a study showing that postoperative radiotherapy prolongs prognosis [28, 29]. On the other hand, one study shows that postoperative chemotherapy and radiotherapy for TNET do not contribute to prognosis [30]. In our study, we added chemotherapy to five of nine patients with TNET before or after surgery and postoperative radiotherapy to nine of 16 patients with thymic carcinoma. Even among high-grade TNET, preoperative chemoradiotherapy enabled complete resection, and postoperative chemotherapy led to a long-term prognosis without recurrence, as shown in Table 2. These results suggested that pre/postoperative additional treatment possibly prolonged the prognosis in TNET or thymic carcinoma. For thymic carcinoma, the combination of paclitaxel and carboplatin is reported to be comparatively effective, as the overall response rate was 22 to 36% for stage IV or recurrent cancer in a phase II trial [31, 32]. In addition to chemotherapy, there appeared some other options. We expect the potential efficacy of new molecular targeted therapies and immune checkpoint inhibitors. Lenvatinib was approved in Japan for the additional treatment of unresectable thymic carcinoma in 2021 [33]. Additionally, immune checkpoint inhibitors have been reported to be effective in recurrent and progressive cases [34, 35]. For TNET, the NCCN guidelines state the efficacy of somatostatin analogs and molecular-targeted drugs [9]. In this study, we used a somatostatin analog, everolimus, only in one case for short-term treatment. There are no large-scale prospective studies regarding perioperative therapy for TNET and thymic carcinoma. Effective additional treatment need to be established, in the future.

### Limitations

This study has several limitations. First, selection bias was inevitable, as this study was a retrospective study with a limited number of cases at a single institution and included only surgical cases. Second, some of our diagnoses and classifications may not be comparable with other studies since lymph node dissection was not systematically performed and the TNET group included one borderline lesion with NET components. Finally, assessment for prognostic factors was based on univariate analysis instead of multivariate analysis. Multivariate analysis was not performed because the number of samples was small and the reliability of the analysis results would be low.

## Conclusion

We investigated TNET and thymic carcinomas that were surgically resected in our department, and there was no statistically significant difference in prognosis between the two types of tumors. Even with high-grade TNET and thymic carcinoma, some patients achieve long-term survival after aggressive multidisciplinary treatment, including surgery. Complete resection may be a valuable treatment in TNET and thymic tumors, although more data are needed.

## Data Availability

No datasets were generated or analysed during the current study.

## Abbreviations

TNET: Thymic neuroendocrine tumors
WHO: World Health Organization
OS: Overall survival
PFS: Progression-free survival
CDDP: cisplatin
VP-16: Etoposide
PR: Partial response
SD: Stable disease

## References

[1] Cardillo G, Rea F, Lucchi M, Paul MA, Margaritora S, Carleo F (2012). Primary neuroendocrinetumors of the thymus: a multicenter experience of 35 patients. Ann Thorac Surg.

[2] Filosso PL, Yao X, Ahmad U, Zhan Y, Huang J, Ruffini E (2015). Outcome of primary neuroendocrine tumors of the thymus: a joint analysis of the international thymic malignancy interest group and the European Society of thoracic surgeons databases. J Thorac Cardiovasc Surg.

[3] Marx A, Shimosato Y, Kuo TT, Chan JK, Travis WD, Wick WR, Travis WD, Brambilla E, Muller-Hermelink HK, Harris CC (2004). Thymic neuroendocrine tumours. World Health Organization Classification of Tumours: Pathology and Genetics of tumours of the lung, Pleura, Thymus and heart.

[4] Travis WD, Brambilla E, Burke AP, Marx A, Nicholson AG. WHO Classification of Tumours of the Lung, Pleura, Thymus and Heart, Fourth Edition. France: International Agency for Research on Cancer.2015;234–242. 10.1097/JTO.0000000000000663 26291007

[5] WHO Classification of Tumours Editorial Board (2021). Thoracic tumours. WHO classification of Tumours. Fifth Edition.

[6] Koga K, Matsuno Y, Noguchi M, Mukai K, Asamura H, Goya T (1994). A review of 79 thymomas: modification of staging system and reappraisal of conventional division into invasive and non-invasive thymoma. Pathol Int.

[7] Huang J, Detterbeck FC, Wang Z, Loehrer PJ (2010). Standard outcome measures for thymic malignancies. J Thorac Oncol.

[8] NCCN Clinical Practice Guidelines in Oncology: Thymomas and Thymic Carcinomas, Version 1. (2022) Online: www.nccn.org [accessed May 20, 2022].

[9] NCCN Clinical Practice Guidelines in Oncology: Neuroendocrine and Adrenal Tumors, Version 1. (2022) Online: www.nccn.org [accessed May 20, 2022].

[10] Suster S, Rosai J (1991). Thymic carcinoma. A clinicopathologic study of 60 cases. Cancer.

[11] Moran CA, Suster S (2000). Neuroendocrine carcinomas (carcinoid tumor) of the thymus. A clinicopathologic analysis of 80 cases. Am J Clin Pathol.

[12] Ströbel P, Zettl A, Shilo K, Chuang WY, Nicholson AG, Matsuno Y (2014). Tumor genetics and survival of thymic neuroendocrine neoplasms: a multiinstitutional clinicopathologic study. Genes Chromosomes Cancer.

[13] Tiffet O, Nicholson AG, Ladas G, Sheppard MN, Goldstraw P (2003). A clinicopathologic study of 12 neuroendocrine tumors arising in the thymus. Chest.

[14] Kondo K, Monden Y (2003). Therapy for thymic epithelial tumors: a clinical study of 1,320 patients from Japan. Ann Thorac Surg.

[15] Weksler B, Holden A, Sullivan JL (2015). Impact of positive nodal metastases in patients with Thymic Carcinoma and thymic neuroendocrine tumors. J Thorac Oncol.

[16] Filosso PL, Yao X, Ruffini E, Ahmad U, Antonicelli A, Huang J, Guerrera F (2016). Comparison of outcomes between neuroendocrine thymic tumours and other subtypes of thymic carcinomas: a joint analysis of the European Society of Thoracic Surgeons and the International Thymic Malignancy Interest Group. Eur J Cardiothorac Surg.

[17] Zhao Y, Gu H, Fan L, Han K, Yang J, Zhao H (2017). Comparison of clinical features and survival between thymic carcinoma and thymic carcinoid patients. Eur J Cardiothorac Surg.

[18] Wen J, Chen J, Chen D, Liu D, Xu X, Huang L (2018). Evaluation of the prognostic value of surgery and postoperative radiotherapy for patients with thymic neuroendocrine tumors: a propensity-matched study based on the SEER database. Thorac Cancer.

[19] Song Q, Zhang LL, Qi Y, Xing KL, Wu XH (2019). Effect of clinicopathologic features on survival of patients with thymic carcinomas and thymic neuroendocrine tumors: a population-based analysis. Curr Probl Cancer.

[20] Bakhos CT, Salami AC, Kaiser LR, Petrov RV, Abbas AE (2020). Thymic neuroendocrine tumors and thymic carcinoma: demographics, treatment, and Survival. Innovations.

[21] Detterbeck F, Youssef S, Ruffini E, Okumura M (2011). A review of prognostic factors in thymic malignancies. J Thorac Oncol.

[22] Sullivan JL, Weksler B (2017). Neuroendocrine tumors of the thymus: analysis of factors affecting survival in 254 patients. Ann Thorac Surg.

[23] Hamaji M, Omasa M, Nakagawa T, Miyahara S, Suga M, Kawakami K (2020). Survival outcomes of patients with high-grade and poorly differentiated thymic neuroendocrine carcinoma. Interact Cardiovasc Thorac Surg.

[24] Song Z, Zhang Y (2013). Primary neuroendocrine tumors of the thymus: clinical review of 22 cases. Oncol Lett.

[25] Fukai I, Masaoka A, Fujii Y, Yamakawa Y, Yokoyama T, Murase T (1999). Thymic neuroendocrine tumor (thymic carcinoid): a clinicopathologic study in 15 patients. Ann Thorac Surg.

[26] Filosso PL, Ruffini E, Solidoro P, Roffinella M, Lausi PO, Lyberis P (2017). Neuroendocrine tumors of the thymus. J ThoracDis.

[27] Takei H, Kondo H, Miyaoka E, Asamura H, Yoshino I, Date H (2014). Surgery for small cell lung cancer: a retrospective analysis of 243 patients from Japanese Lung Cancer Registry in 2004. J Thorac Oncol.

[28] Cardillo G, Treggiari S, Paul MA, Carleo F, De Massimi AR, Remotti D (2010). Primary neuroendocrine tumors of the thymus: a clinico pathologic and prognostic study in 19 patients. Eur J Cardiothorac Surg.

[29] de Montpréville VT, Macchiarini P, Dulmet E (1996). Thymic neuroendocrine carcinoma (carcinoid): a clinicopathologic study of fourteen cases. J Thorac Cardiovasc Surg.

[30] Filosso PL, Yao X, Ahmad U, Zhan Y, Huang J, Ruffini E, European Society of Thoracic Surgeons Thymic Group Steering Committee (2015). Outcome of primary neuroendocrine tumors of the thymus: a joint analysis of the International Thymic Malignancy Interest Group and the European Society of thoracic surgeons databases. J Thorac Cardiovasc Surg.

[31] Lemma GL, Lee JW, Aisner SC, Langer CJ, Tester WJ, Johnson DH (2011). Phase II study of carboplatin and paclitaxel in advanced thymoma and thymic carcinoma. J Clin Oncol.

[32] Hirai F, Yamanaka T, Taguchi K, Daga H, Ono A, Tanaka K (2015). A multicenter phase II study of carboplatin and paclitaxel for advanced thymic carcinoma: WJOG4207L. Ann Oncol.

[33] Sato J, Satouchi M, Itoh S, Okuma Y, Niho S, Mizugaki H (2020). Lenvatinib in patients with advanced or metastatic thymic carcinoma (REMORA): a multicenter, phase 2 trial. Lancet Oncol.

[34] Giaccone G, Kim C, Thompson J, McGuire C, Kallakury B, Chahine JJ (2018). Pembrolizumab in patients with thymic carcinoma: a single-arm, single-center, phase 2 study. Lancet Oncol.

[35] Cho J, Kim HS, Ku BM, Choi YL, Cristescu R, Han J (2019). Pembrolizumab for patients with refractory or relapsed thymic epithelial tumor: an open-label phase II trial. J Clin Oncol.

